# Fæcal Fistulæ and Artificial Anus

**Published:** 1895-07

**Authors:** J. E. Thompson

**Affiliations:** Texas Medical College; Galveston


// Texas Medical Journal.

ESTABLISHED JULY, 1885.

Published Monthly.—Subscription $2.00 a Year.

Vol. XI. AUSTIN, JULY, 1895. No. 1.

### Original Contributions.

For Texas Medical Journal.

### FÆCAL FISTULÆ AND ARTIFICIAL ANUS.

BY PROF. J. E. THOMPSON, M. D., F. R. C. S., TEXAS MEDICAL COLLEGE.

[Read at the Dallas meeting, T. S. M. A., April 23, 1895.]

HAVING had under my care, during the past few years, a number of cases of fæcal fistula and artificial anus, I thought it might interest the members of this Association to know the methods I employed to overcome this disagreeable and dangerous condition. I shall, however, not confine myself to treatment, but shall first give some reasons, which I feel to be all powerful, to prove to your satisfaction that we do not establish an artificial anus as often as we ought to.

In cases of insurmountable intestinal obstruction, no one would hesitate to open the gut above the constriction, evacuating the contents, and establishing an artificial anus. We ought, however, to go further than this and make an artificial anus in cases where the obstruction is surmountable, if the intestine above is immensely distended, and particularly where we have peritonitis associated with this condition.

In acute diffuse peritonitis, resulting from perforation or traumatism, one of the chief factors to deal with, apart from the infection of the great serous sac, is the enormous distension of the intestine which results from paralysis of the muscular walls of the gut. *Through this paralyzed wall, it has been proven, that the bacillus coli and other organisms can pass, thus keeping up the supply of virulent material in the already infected cavity. It is a little doubtful which organism is the main cause of peritonitis resulting from perforation, for Lockwood† seems to think that the bacillus coli is usually found, because it is easier to grow than the others, and not because it is more frequently present. However this may be, the fact remains that distended gut is a great danger in itself, in that it allows these organisms to pass through in enormous quantities. To avoid this the intestine must be opened and the contents evacuated. Sometimes, and particularly in cases of mere distension, unassociated with perforation resulting from some obstruction, a simple incision, which is afterwards stitched up with Lembert's sutures, suffices; but where the gut is very distended, and where we have acute peritonitis, it is better to open a distended coil of ileum, choosing a spot as near the cæcum as possible, by a short incision, and attach the edges of the opening to the skin by three or four silk sutures. The gut should be washed with sterilized water, and the contents thoroughly evacuated.

The first to advise this as a definite procedure in cases of acute diffuse peritonitis, was Henrotin, of Chicago,‡ who, after operating for an acute appendicitis in a boy, found that the dangerous symptoms continued until the third day, when the ligature on the stump of the appendix gave way, and a sudden gush of fæcal fluid resulted in immediate relief of all the symptoms.

He carried this into practice on a second case of appendicitis with marvelous success. Pus was evacuated from around a matted appendix, which was left untouched, and the abdominal cavity washed out with saline solution. The ileum and cæcum were enormously distended, so the cæcum was incised, and the edges attached, by a few silk stitches, to a slit made in the abdominal wall in the iliac region. He attributes the recovery of this patient to the establishment of an artificial anus.

§Hadra advised this procedure and practiced it on one of his patients with good results, and I had occasion to make an artificial anus on his patient for the same condition.

Recently Lockwood || has reported three cases of acute diffuse

---

* Treves, Lettsomian Lectures, 1894.

† Lockwood, London Lancet, March 2, 1895.

‡ American Journal of Obstetrics, 1893, p. 100.

§ Hadra, New York Medical Journal, June 2, 1894.

|| Lockwood, London Lancet, October 27, 1894, p. 975.

peritonitis cured by operation, in one of which he attributed the successful result to the accidental formation of a fæcal fistula.

The after-treatment of an artificial anus so made is easy in the extreme, particularly as we know the condition of the intestine and the relation of the loop of gut to its surroundings. The following case (I) will indicate the method of procedure:

CASE I.—Miss O. had previously been operated on by Dr. Hadra for peritonitis, and an artificial anus made. Symptoms of intestinal obstruction above the artificial anus supervened, and peritonitis set in with high temperature, tympanites and vomiting. I opened the abdomen above the umbilicus, cleaned out the cavity, seized a distended coil of gut, which I opened and stitched to the side of my abdominal incision. Immediate relief followed the profuse fæcal flow, and the symptoms disappeared.

Some weeks afterwards, by washing out the intestine, I managed to re-establish the intestinal channel, and determined to close up the artificial anus I had made. An elliptical incision was made around the artificial anus, and the skin raised towards the opening and clamped. The incision was then deepened alongside the opening, and the peritoneal cavity opened. The incision was then deepened all around the anus, and plugs of iodoform gauze successively inserted around the loop of gut until the intestine, with an elliptical piece of abdominal wall, was lifted directly into the field of operation. The abdominal wall was now dissected from the gut, the edges of the opening trimmed wlth scissors, and the slit closed with two rows of Lembert's sutures. The intestine was then dropped into the peritoneal cavity, which was closed.

The patient recovered from the operation, and fæcal matter began to discharge from the lower artificial anus. I proposed to close this also, when obstruction set in again, and the patient's friends refused further interference.

Many methods have been used to cure artificial anus without interfering with the peritoneal cavity, and although many have been credited with great success, failures have been only too common.

Some varieties have a spontaneous tendency to heal, and this holds particularly for fistulæ connected with the vermiform appendix and those resulting from operations on the pelvic organs, where a long sinus extends down to a small opening in the bowel. Probably, also, fistulæ, connected with the large bowel, have a greater tendency to heal than those connected with the small gut. The main hindrance to the healing process is the formation of a spur or éperon, formed by the concave wall of the gut, which sticks into the artificial opening, dividing it into two parts, one communicating with the proximal end of the gut, and the other with the distal. This spur acts as a valve, directing the fæcal flow through the artificial anus, instead of allowing it to pass along the gut.

Dupuytren devised an instrument, called an enterotome, which clamped the two sides of the spur and caused adhesion of the peritoneal surfaces. This fell into disuse on account of fatal cases, where ulceration into the peritoneal cavity resulted, but lately it was revived by other surgeons, who modified and improved it. The best enterotome is probably Gross's, which divides and removes the projecting spur. It consists of modified torsion forceps, the points of which are made in the form of two circular opposing rings. *Herman's statistics of this method show out of 84 cases a mortality of 8.5 per cent., a complete cure of 50 cases, and in 26 considerable improvement.

Banks attempted to overcome the spur by the ingenious method of inserting a piece of strong rubber tubing into the proximal and distal ends of the gut.

The constant tendency of this piece of rubber to straighten itself exerted in some cases sufficient pressure on the spur to obliterate it.

In cases where there was no spur, attempts have been made to close the opening by various plastic operations (as had been employed in case 2), or occasionally by pressure from an elastic truss, or again, by stimulating the granulations with nitrate of silver or some other caustic, and approximating them with strapping, or hair lip pins. All these methods have cures accredited to them, but usually they are quite ineffectual.

The following case shows how ineffectual all these sample methods may be and how easy it is to close up an opening where the spur is practically absent.

CASE 2.—G. T., age 15, male, was sent to me from the interior of the State, with a history of appendicitis and abscess formation, the evacuation of which had left a fæcal fistula. Operation had been attempted twice previously and consisted of vivifying the edges of the wound and drawing them together with

*London Medical Record, 1883, p. 187.

wire sutures. Both had been signal failures and the last attempt had been followed by an erysipelatous inflammation of the abdominal wall.

On examination the patient showed an opening above and a little internal to the right anterior superior iliac spine, which discharged fæcal matter profusely; and around the edges were a number of sinuses communicating with the wound. As the abdominal wall was unhealthy, I decided on performing an anastomosis between the termination of the ileum and the transverse colon, thus shutting off the ascending colon from the fæcal current. This was done without the aid of plates, through a median subumbilical incision. The boy recovered quickly from the operation, but fæcal matter continued to discharge in small quantities from the artificial anus, and at the end of a month, the patient becoming impatient I attacked the fæcal fistula directly. The operation was conducted on the same lines as case 1, was very easy and resulted in a perfect cure. During the operation the caput cæcum coli and vermiform appendix were found healthy, and the fistulous opening found to be in the commencement of the ascending colon.

In such cases, I think, the intestine should always be freely exposed, the opening sutured and the gut dropped back. But in certain cases there may have been such destruction of the intestinal wall that suture is an impossibility without materially diminishing the size of the canal, and in such the bowel must be resected and reunited. †Makin has recorded a number of cases of enterectomy for artificial anus, which give the following analysis: Out of 39 cases, 15 (38.4 per cent) died; [9 from septic peritonitis; 5 from fæcal extravasation, in 3 of which it was from the mesenteric border]; 24 recovered, 3 of which were left with an artificial anus.

His method of procedure is precisely the same in the early stages as that described in case 1, and all precautions are taken to prevent extravasation of fæcal contents. Then the bowel with the fistula in it is cut away with scissors beyond the site of the old adhesions and suture of the divided ends proceeded with.

There is a class of cases, which, unfortunately, is only too common, viz.: perforating ulcers of the vermiform and appendix and cæcum, which occasionally result in a fæcal fistula of a permanent character, situated in the right iliac region. These cases

---

†St. Thomas Hospital Reports. 1884.

usually come under our observation as a sinus, discharging more or less pus with fæcal matter, and with a history pointing to an abscess probably associated with an appendicitis. The history is often vague, but occasionally it may be clear and concise.

Operations for the relief of this condition are extremely difficult; and one can never tell beforehand, whither one may be led in the attempt to locate the opening in the diseased appendix. Usually it remains imbedded firmly in dense adhesions, and may be closely glued to the iliac vessels, and even to the ureter, rendering any attempt to resect or close up the opening in the gut an impossibility. Case 3 was of this nature and removal of the diseased appendix post-mortem was only accomplished by wounding the iliac vessels.

Southam* was, I believe, the first to suggest shutting off the infected loop of bowel by establishing an anastomosis between the termination of the ileum and the transverse colon. This he successfully carried into practice on one of his patients, the fistula healing up in a few weeks. I employed this method in Case II, but afterwards attacked the fistula by the direct method. In the following case, the operation terminated fatally from shock.

CASE III.—E. S., male, age 30. Came under my care with three sinuses in the right groin just above Pouparts' ligament. He gave an obscure history of abscess formation some few years back. On examination no fæcal discharge could be discovered, but the patient assured us that a solution of potassium permanganate injected into the sinus came out of the rectum. This test however failed us. I decided to explore the sinuses, and found the iliac fossa occupied by a pus sac, matted intestine forming the anterior wall. Near the brim of the pelvis in close proximity to the sacro-iliac joint, a mass of fixed adhesions was found. No dead bone was discovered. The cavity was thoroughly scraped out, injected with iodoform emulsion and absolutely closed. The sutures broke down in a few days and fæcal matter came away in great quantity.

A diagnosis of old appendicitis was made, but, as the patient's condition was very unfavorable, no operation was thought of. Three months later, at the patient's earnest request, I established an intestinal anastomosis (without plates) between the ileum and the transverse colon. Unfortunately, our patient never recovered from the shock of the operation, but died the same day.

---

* Lancet, London, Oct. 8, 1892.

The post-mortem examination revealed the appendix almost unrecognizable in a mass of adhesions, firmly united to the iliac vessels. A small opening near its base passed into the interior of the abscess cavity.

The following case I attacked directly, as it seemed probable that the appendix lay in front of the cæcum:

CASE IV.—J. A., male, age 26. A clear history of suppuration, due to perforation of the vermiform appendix two years ago. The abscess cavity had been opened and the resulting sinus had discharged feculent pus ever since.

I operated by an elliptical incision embracing the sinus, opened the abdomen and proceeded to dissect out the appendix from a mass of adhesions, which were almost cartilaginous in their density. I inadvertantly opened the ascending colon, which was closed with Lembert's sutures, and, after great difficulty, succeeded in finding a perforation in the cæcum, situated just at the base of the appendix in the orifice of which lay a small fæcal concretion which had failed to come out of the fistula. The appendix was removed, the perforation excised and the resulting hole closed by two rows of continuous Lembert's sutures. The fistulous track was then excised together with a large area or inflamed and infected abdominal wall. The cavity was then packed with iodoform gauze.

The patient rallied quickly, but the wound discharged freely, and even now is not entirely healed up, owing to the extrusion from time to time of silk sutures which were used to unite the abdominal muscles. However, no fæcal matter has passed, and I think the prognosis is favorable.

The difficulties of operations of this nature can not be adequately described; they are so numerous that only experience can teach us how to deal with them. It is better, if possible, not to open up the general peritoneal cavity, but usually one finds it impossible to avoid this, and as it is rarely that one can thoroughly disinfect the fistulous tract, it is much better to pack the wound with iodoform gauze, to secure drainage, than to run the risks of peritonitis.

* Dudley reports a case of this nature where there was a clear history of appendicitis, the operation for removal being followed by a fæcal fistula discharging almost all the bowel contents.

He operated and found an intact appendix, but five perfora-

* Amer. Journ. of Obstetrics. 1892, p. 145.

tions in the cæcum near the ileum, each the size of a lead pencil. He closed each opening with silk, and closed the abdomen. His patient progressed favorably for thirty-six hours and then died of perforative peritonitis, owing to the stitches giving way.

Another class of fæcal fistula resulted from malignant disease of the intestine, in which perforation has occurred and the resulting abscess has penetrated the abdominal wall. I record the history of two such cases which will give a fair idea of the condition under consideration.

CASE V.—A. H., age 29, male. Suffering from a fæcal fistula in the left iliac region. The history was obscure; no intestinal obstruction, and his youth rather discredited a diagnosis of malignant disease. An abscess had formed some months previously, and after being opened had discharged fæcal matter almost continually.

On exploring the fistulous track, I found that it led to the sigmoid flexure, passing into the bowel by a small opening. The colon was found to be the seat of a malignant growth fully four inches long. The operation was abandoned, and the patient died two months afterwards.

† CASE VI. Sarcoma of ileum, perforation, localized peritonitis, evacuation of contents of abscess cavity, drainage, death.

This case came under my care with a large suprapubic swelling, evidently due to a localized peritonitis. I made a median incision and evacuated the purulent fæcal contents of a cavity whose walls consisted of matted intestine. The cavity was drained and irrigated, and the patient lived about three weeks. The post-mortem examination revealed a sarcoma of the ileum with a perforation in its wall.

In these cases no treatment short of excision of the gut would be of any avail. In Case V, this would have been possible if the patient's condition would have allowed such a procedure. In Case VI, it would have been an utter impossibility, owing to the size and extent of the growth.

Fæcal fistulæ following operative procedures on the pelvic organs I have purposely avoided, as the field is too vast for the present paper, but those who wish to study this subject will be amply repaid by a careful perusal of the above quoted papers by Dudley, where a complete resumé of the work done in this department is given.

---

† Reported in extenso in Texas Medical Journal, April, 1893.

